# Image guidance and positioning accuracy in clinical practice: influence of positioning errors and imaging dose on the real dose distribution for head and neck cancer treatment

**DOI:** 10.1186/s13014-018-1141-8

**Published:** 2018-10-01

**Authors:** Katharina Bell, Norbert Licht, Christian Rübe, Yvonne Dzierma

**Affiliations:** Department of Radiotherapy and Radiation Oncology, Saarland University Medical Centre, Kirrberger Str. Geb. 6.5/Saar, D-66421 Homburg, Germany

**Keywords:** IGRT, Positioning accuracy, NTCP modelling, Kilovoltage imaging, Megavoltage imaging, Treatment margins

## Abstract

**Background:**

Modern radiotherapy offers the possibility of highly accurate tumor treatment. To benefit from this precision at its best, regular positioning verification is necessary. By the use of image-guided radiotherapy and the application of safety margins the influence of positioning inaccuracies can be counteracted. In this study the effect of additional imaging dose by set-up verification is compared with the effect of dose smearing by positioning inaccuracies for a collective of head-and-neck cancer patients.

**Methods:**

This study is based on treatment plans of 40 head-and-neck cancer patients. To evaluate the imaging dose several image guidance scenarios with different energies, techniques and frequencies were simulated and added to the original plan. The influence of the positioning inaccuracies was assessed by the use of real applied table shifts for positioning. The isocenters were shifted back appropriately to these values to simulate that no positioning correction had been performed. For the single fractions the shifted plans were summed considering three different scenarios: The summation of only shifted plans, the consideration of the original plan for the fractions with set-up verification, and the addition of the extra imaging dose to the latter. For both effects (additional imaging dose and dose smearing), plans were analyzed and compared considering target coverage, sparing of organs at risk (OAR) and normal tissue complication probability (NTCP).

**Results:**

Daily verification of the patient positioning using 3D imaging with MV energies result in non-negligible high doses. kV imaging has only marginal influence on plan quality, primarily related to sparing of organs at risk, even with daily 3D imaging. For this collective, sparing of organs at risk and NTCP are worse due to potential positioning errors.

**Conclusion:**

Regular set-up verification is essential for precise radiation treatment. Relating to the additional dose, the use of kV modalities is uncritical for any frequency and technique. Dose smearing due to positioning errors for this collective mainly resulted in a decrease of OAR sparing. Target coverage also suffered from the positioning inaccuracies, especially for individual patients. Taking into account both examined effects the relevance of an extensive IGRT is clearly present, even at the expense of additional imaging dose and time expenditure.

## Introduction

Modern techniques in radiotherapy offer a more and more precise application of the dose to the target volume. This allows for an adequately sparing of the surrounding tissue while the tumor can be covered with dose as accurately as possible.

An exactly reproducible patient positioning is a prerequisite for the treatment to be successful, as any shift relative to the planned position can result in an underdosage of the target volume or an overdosage of the surrounding organs at risk (OAR). Beside the application of positioning and localization facilities like thermoplastic masks or laser marks, potential inter- and intrafractional shifts are already considered in the planning process by applying safety margins to expand the clinical target volume (CTV) to the planning target volume (PTV). Taking into account the systematic and random errors, there are several recipes to determine the width of these margins [1–3]. The more precisely patient positioning can be achieved the smaller can safety margins be chosen. At the same time OARs can be better spared using narrower margins, so exact positioning results in better sparing of OARs while offering the same target coverage.

To put this into effect, frequent positioning verification before treatment in the context of Image Guided Radiotherapy (IGRT) is the nowadays standard. Modern linear accelerators are equipped with different imaging modalities that can be applied in treatment position. However, as these modalities often use ionizing radiation, the accurate position verification implies a burden of an additional dose, which depends on the imaging energy, number of projections and frequency of the verifications. Ideally, the patients´ position should be controlled and corrected daily, however, the additional dose of the imaging cannot generally be neglected.

Hence there is a trade-off between two effects: By verifying the patients´ set-up regularly the positioning accuracy can be increased, undesirable dose smearing can be avoided and the CTV-PTV expansion margins can be decreased. However, the additional dose may also have a negative effect on the total dose.

The aim of this study is to examine these two effects. To analyze the effect of the additional imaging dose on the plan quality different realistic and representative imaging scenarios are simulated and the imaging dose is added to the original treatment plan, respectively. Within these scenarios we differentiate between imaging techniques (planar vs. 3D), different energies (kV vs. MV) and daily vs. non daily set-up verification. The effect of potential positioning uncertainties on the real dose distribution is also analyzed by simulating different scenarios with variable numbers of set-up corrections. So it can be investigated if the advantage of a higher precision with the possibility of steeper dose gradients and smaller margins predominates over the disadvantage of the additional dose.

For both effects an evaluation of the dosimetrical plan quality is performed as well as modelling biological aspects in terms of normal tissue complication probabilities. These theoretical estimations are performed on an individual basis for a realistic collective of head and neck (H&N) cancer patients for best possible transferability into the clinical routine. H&N is one of the main indications for regular set-up verifications as the close vicinity of the OARs to the target volume and its complex shape requires steep dose gradients and an exact patient positioning [4, 5].

This is the first study to combine and balance both described aspects regarding IGRT. The current literature offers a number of studies either focusing on the additional imaging dose or the influence of positioning uncertainties on the dose distribution. Moreover, most studies regarding imaging dose primarily concentrate on the dose by itself [6–13], only few have the focus on the clinical consequences for plan quality [14–18]. There is one study to evaluate systematically the influence of different imaging scenarios on plan quality, however, this study deals with prostate treatment [19]. The effect of dose smearing is also sparsely examined by now, most studies on this topic rely on rather theoretical models of the average positioning errors and the resulting dose volume histogram [1, 3, 20–23]. So to our knowledge there is no such systematic investigation considering the clinical effect of imaging dose together with the effect of potential dose smearing, for both dosimetrically plan quality and biological aspects.

## Material and methods

### Collective and equipment

The analysis for this study was performed for a collective of 40 patients with head-and-neck cancer, treated at our institution in 2013. Several indications are included, the collective mostly consists of patients with pharyngeal cancer, cancer of the mouth- or base of the tongue, sporadically also tonsil, parotid and larynx.

The 40 patients were treated with a total of 1325 fractions, with 20–60 fractions per patient depending on indication and concept. Most patients received 30–35 treatment fractions. The prescribed dose to the PTV generally was 50 Gy (2 Gy daily) using a 7–13 beams IMRT with 6 MV photons. Partly, from a dose of 30 Gy, the single fraction dose had been changed from 2 Gy daily in one fraction to 1.4 Gy with two fractions per day following a hyperfractionated concept. With a similar number of beams the treatment plans of this collective include one or two boosts up to a total dose of 60–70 Gy. The CTV-PTV margin amounts to about 5–10 mm.

Planning was performed in the Pinnacle TPS V9.2 on the basis of planning CTs acquired with a Philips Brilliance Big Bore 120 kV.

At our department, three Siemens linacs, two Artistes and one Oncor, with different imaging modalities are available. All three machines can perform imaging using the 6 MV treatment beam line (TBL), the two Artistes are additionally equipped with a dedicated image beam line (IBL) with a nominal energy of 1 MV [24, 25]. A kV modality using an additional X-ray tube is installed at one of the two Artiste machines. Due to the different energies of the imaging modalities, the particular imaging dose differs, just as the image quality.

Imaging is individually performed as prescribed by the radiotherapist, also depending on the current workflow and schedule of the clinical routine. For immobilization, patients are positioned using thermoplastic masks, considering the room lasers to align with corresponding marks. The performed verification images are compared with the digitally reconstructed radiographs or the planning CT and positioning errors are corrected on-line with no action level.

### Evaluation of the additional imaging dose

To analyze the effect of the additional imaging dose on the plan quality, the appropriate energies and beam properties of the imaging modalities need to be modelled and commissioned in the TPS. For our modalities this has already been realized in former studies [26, 27]. Additionally to the 6 MV treatment beam line, the IBL and kV imaging have been included in the TPS, so that the distribution of the imaging dose can be calculated and added to the original treatment plan for every patient.

As in our institution the kind and frequency of the executed set-up verifications depend on medical decisions and clinical workflow, patients receive different IGRT schemes. For H&N cancer patients imaging is generally not done daily, but about every third fraction.

To examine the influence of the imaging dose systematically for the different IGRT modalities, we simulated the following hypothetic scenarios (Table 1):

**Table 1:** Imaging scenarios

Scenario 1	Original plan: without imaging dose
Scenario 2	Not daily: actual performed imaging
Scenario 3	Daily: kV CBCT 200°
Scenario 4	Daily: IBL CBCT 200°
Scenario 5	Daily: 1xTBL CBCT, 4× TBL planar images per week

Scenario 1 represents the original treatment plan without imaging dose, as it was accepted for treatment. This serves as a reference for the comparison with the remaining scenarios.

For scenario 2 we consider the imaging dose every patient received in reality by their individual IGRT schedule. We obtained this data retrospectively from the Record and Verify (R&V) system. This scenario illustrates the non-daily but real imaging.

As it is desirable to verify the patients’position for every fraction, daily imaging is simulated in scenarios 3–5 for the different energies and techniques.

Scenario 3 contains the dose distribution for daily 3D imaging within the kV range. For this collective we consider CBCTs with a 200° rotation. For the kV modality an auto-exposure technique is available, so the mAs product is adapted individually to the patient by a “pre shot”. For the calculation of the imaging beams the mean mAs value of the whole collective is used, which comes to a value of 112mAs per CBCT for this collective.

Analogue to this, scenario 4 implies daily 200° CBCTs for the IBL. For the calculation of the beams for this energy we use monitor units instead of the mAs product. The deposited IBL protocols apply about 6 MU for one H&N 200° CBCT.

Daily 3D imaging with the TBL entail a much too high additional dose, so for the 6 MV energy we choose a scenario (scenario 5) which considers one CBCT per week (200°, 7 MU) and planar images for the remaining days. Planar images are taken with gantry positions of 0° and 90° with 1 MU each.

### Evaluation of the dose smearing

Positioning corrections are performed by shifting the table in the three spatial directions, anterior-posterior, left-right and superior-inferior. The values of these table shifts are also documented in the R&V system.

To analyze the influence of potential positioning errors, the isocenter was shifted back in the original treatment plan appropriately to the applied table shifts to simulate that no positioning correction had been done. The plans were recalculated for every single fraction and summed to a new plan, again considering different scenarios.

Firstly we consider an extreme scenario (extreme plan), in which only plans with shifted isocenters are summed and weighted equally. This simulates, that the patient’s position would never be adapted and no verification imaging would be done. For example, for a patient with 10 verification images, 10 plans with isocenter shifts appropriate to the applied table shifts were recalculated, weighted with a factor of 0.1 respectively and summed up to a new plan. In a previous study we found that the applied table shifts are roughly Gaussian, so we can assume that these 10 fractions are representative for the remaining fractions without imaging [20].

Scenario two represents a more realistic case (realistic plan) where all fractions are considered. For those days on which positioning verification was performed, we can suppose that the patient’s position was corrected and matches with the planning CT, so the original treatment plan is considered. Of course, this is an idealized assumption, minor positioning errors are expected even with image guidance. For all fractions without imaging we apply positioning errors observed on the other days and the previously calculated extreme plan is used. If a patient receives 30 fractions with 10 positioning verifications, the original plan is weighted with a third and the extreme plan with two thirds.

For scenario three (imaging plan) we combine the two examined effects to provide an inside into what would occur in reality. The additional imaging dose of each patient is added to the realistic plan calculated for scenario two. So for this scenario we consider the dose smearing caused by positioning errors as well as the actual imaging dose, individually for each patient.

For all scenarios the recalculation of the plans with shifted isocenters was carried out in the TPS, however, for the summation and further evaluation the single plans were exported in DICOM format and imported into the MATLAB- based Computational Environment for Radiotherapy Research (CERR) [28].

### Assessment of plan quality

For both effects (additional imaging dose and dose smearing) plan quality was first analyzed visually by considering the dose distributions and dose-volume-histograms (DVH).

Sparing of OARs was assessed quantitatively on the base of clinically relevant DVH objectives (Table 2).

**Table 2:** Planning criteria (only valid, where the organ is not inside the PTV)

Parotid glands	Mean < 25Gy; V20Gy < 60%
Spinal chord	D2% < 42Gy
Larynx	D2% < 63Gy; Mean < 44Gy
Vocal chords	D2% < 25Gy

It is examined how many times these acceptance criteria failed with IGRT when passed by the original plan. Moreover, normal tissue complication probability was modelled regarding biological endpoints for particular toxicities (Table 3).

**Table 3:** Endpoints and paramters for the NTCP modelling [29, 30]]

Organ	Endpoint	m	n	Dose50 [Gy]
Parotid glands	Xerostomia	0.18	0.7	64.0
Spinal chord	Myelitis/Necrosis	0.175	0.05	66.5
Larynx	Edema	0.16	0.45	64.3

The different parameters could be entered into the TPS and CERR and the NTCP value could be calculated using the Lymann-Kutcher-Burrmann model (LKB) [31].

$$ NTCP=\frac{1}{\sqrt{2\pi }}\underset{-\infty }{\overset{u}{\int }}\exp \left(\frac{-\mathrm{t}2}{2}\right) dt $$

$$ u=\frac{D-{TD}_{50}(V)}{m\bullet {TD}_{50}(V)} $$

$$ \mathrm{with}\ {TD}_{50}(V)={TD}_{50}(1)/{V}^n $$

*TD*_50_(1) is the dose to the total organ which entails 50% complication risk, *TD*_50_(*V*) is the tolerance dose for a partial volume V, m is the slope of the sigmoidal curve, n describes the volume effect and D is the maximal dose to the organ.

For the effect of dose smearing, target coverage is also a point that needs to be checked by different measures of quality. The homogeneity index is calculated as

$$ HI=\frac{D2\%-D98\%}{D50\%} $$

where Dx% is the dose received by x% of the volume of the PTV.

The amount of under- and overdosage can be assessed by the underdose- and overdose rate:

$$ OR=\frac{TV_{PIV}}{PIV} $$

$$ UR=\frac{TV_{PIV}}{TV} $$

where *TV* denotes the volume of the target, *PIV* is the volume receiving the prescribed dose, and *TV*_*PIV*_ is the volume of the target covered by the prescribed dose (95%). Paddick’s conformity index [32] is given by

$$ CI= OR\bullet UR $$

All these metrics are evaluated for the structure that receives the prescribed total dose (the boost in most cases) as this is most relevant for target coverage. The dose difference between the shifted plans and the original plans is also assessed for each scenario. With the constructed difference-plans it is possible to identify those points where dose deviations exceed a given level (e.g., 1%, 2%, 3%). In a way, this metric is similar to the gamma index pass rate, but disregarding the distance to agreement criteria (which would not make sense when evaluating the effect of spatial shifts).

For the statistical analysis all scenarios were pairwise compared using the Wilcoxon signed-rank test. The calculations were carried out in the Origin Pro 2015 software, taking a value of *p* < 0.05 as significant.

## Results

### Imaging dose

**Fig. 1:**
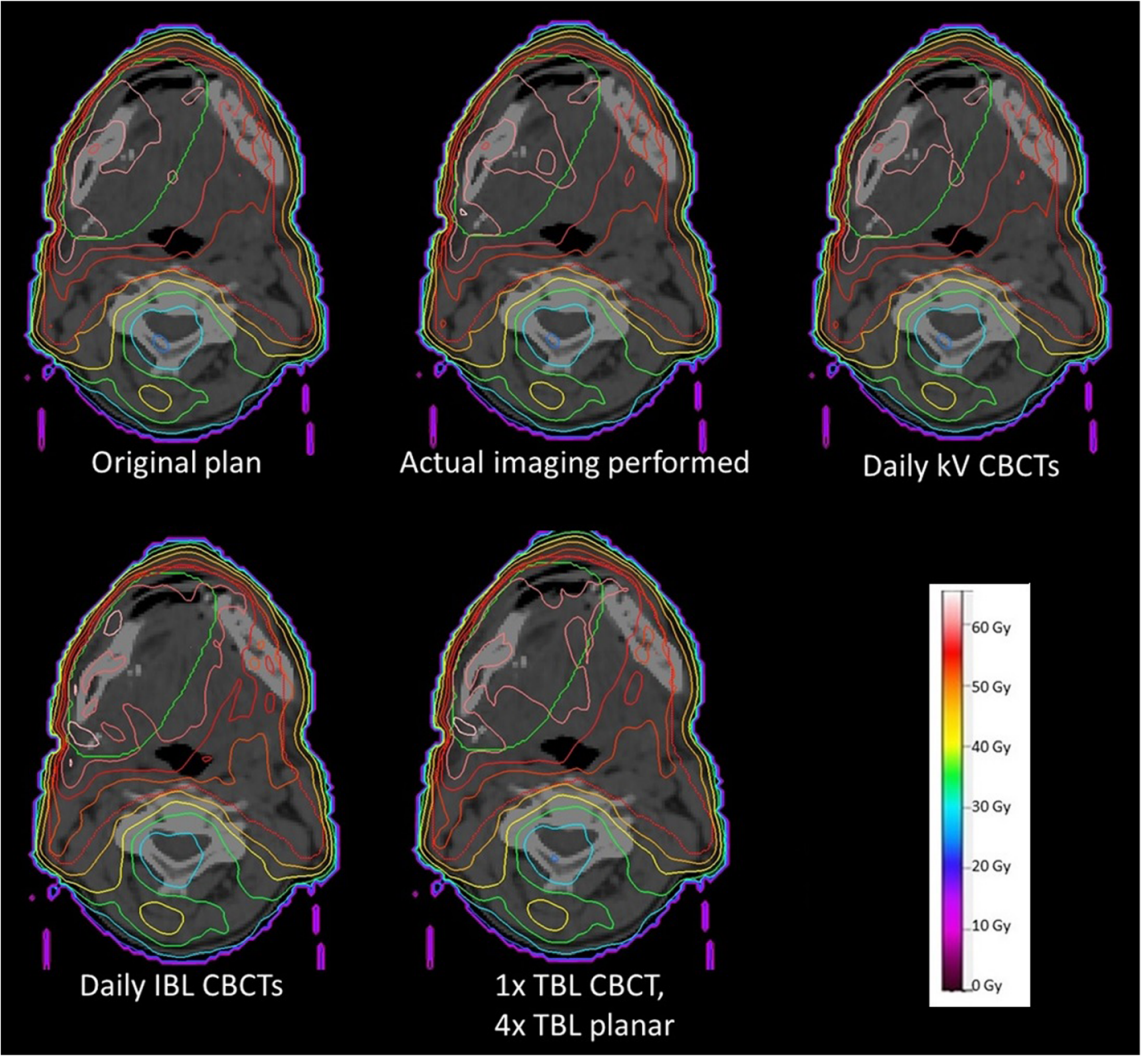
Example Dose distributions of one patient for all scenarios

In Fig. 1 the dose distributions of all 5 imaging scenarios are illustrated for one patient. The tumor of this patient is located at the base of the tongue, the prescriped dose of 60 Gy is separated to a PTV of 50 Gy and a sequential boost of 10 Gy. The actual imaging scheme for this patient contains IBL CBCTs for 4 fractions, IBL planar images for 7 fractions and for one fraction planar images with 6 MV. The additional dose is reflected in small variations of the isodose lines, the 100% isodose spreads over the target volume according to the different scenarios. However, the visual inspection of the dose distribution shows only minor differences.

**Fig. 2:**
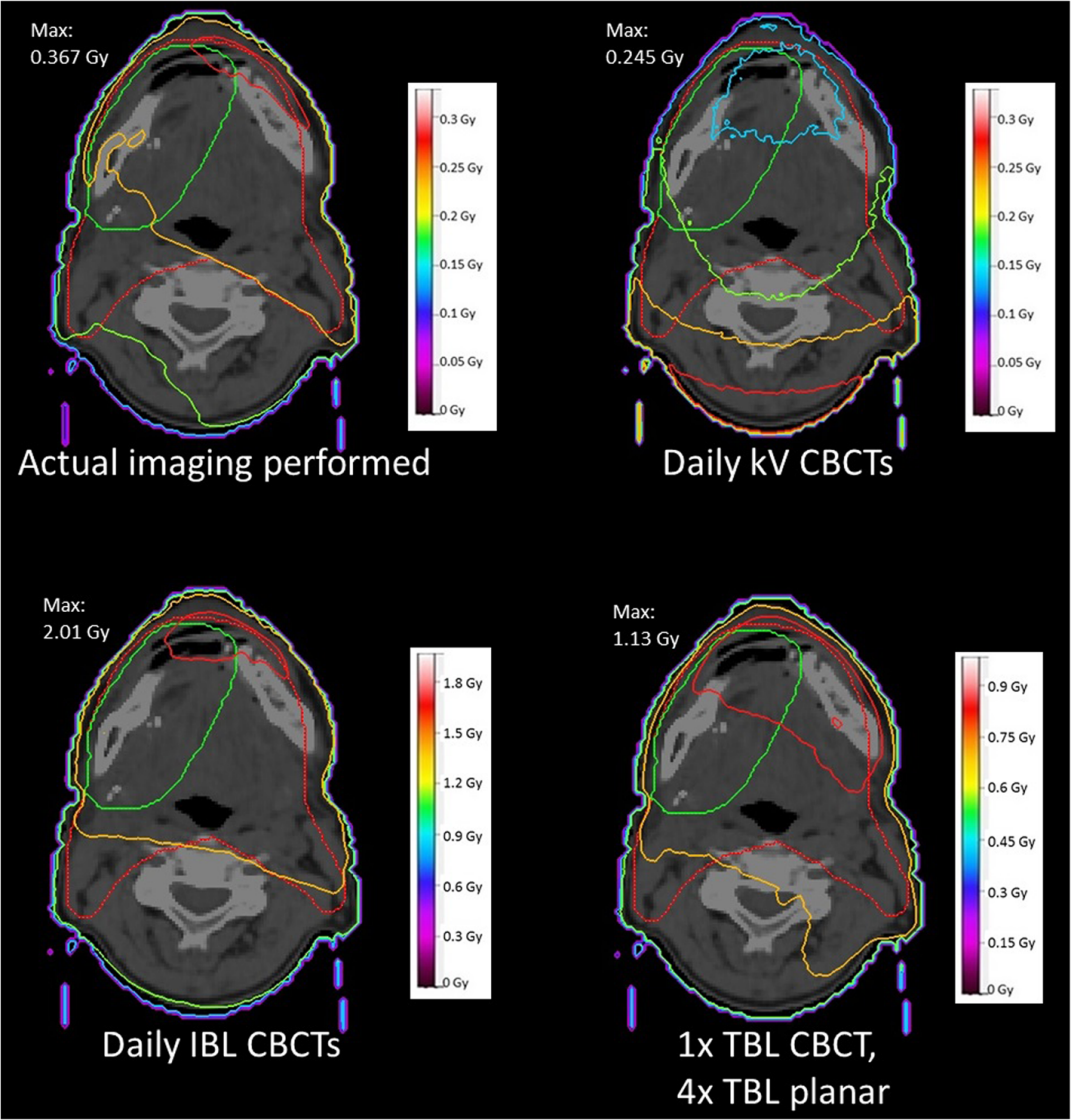
Example Dose distributions of only the imaging doses without the treatment plan for the same patient as in Fig. 1

Figure 2 shows the dose distributions of only the imaging doses for the four scenarios without the treatment plan. It is conspicuous, that the course of the isodoses for the daily kV CBCTs differs from the others. As the X-Ray tube is located opposite to the treatment head, it rotates below the back of the patients.

**Fig. 3:**
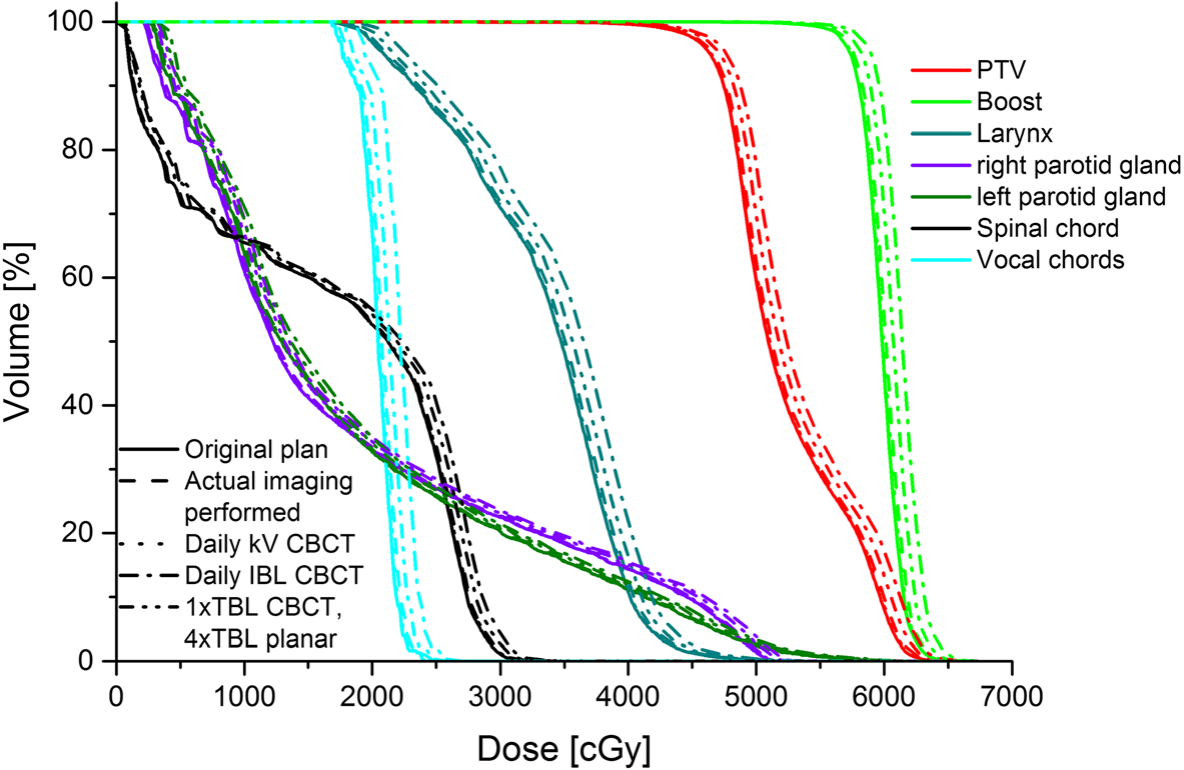
Example DVH of the same patient as in Fig. 1 for all scenarios

To analyze the influence of the additional imaging dose especially to the sparing of OAR, the DVHs of all scenarios for the same patient are presented in Fig. 3.

Scenario 2 and 3 reveal only small differences in comparison to the original plan, the curves almost run congruent. The 6 MV scenario shows a visible effect of the additional imaging dose. However, in comparison to a daily imaging with IBL CBCTs this effect is very slight, although daily MV imaging is used. Scenario 4 results in a marked shift of the curves to higher doses.

Figure 3 illustrates that the additional imaging dose causes an increase of the volume receiving a particular dose. For several patients this causes the DVH constraints for plan acceptability given in Table 2 to be exceeded. Table 4 lists the number of these exceedings for the different scenarios.

**Table 4:** Number of exceedings of the considered planning criteria

Organ	Criteria	Original plan	Scenario 2	Scenario 3	Scenario 4	Scenario 5
Spinal chord	D2% < 42Gy	0	0	0	0	0
Parotid glands	Mean < 25Gy	4	5	4	12	8
	V20Gy < 60%	0	0	0	3	1
Larynx	D2% < 63Gy	2	2	2	4	4
	Mean < 44Gy	0	0	0	1	0
Vocal chords	D2% < 25Gy	2	2	2	8	4
Total		8	9	8	28	17

For scenario 2 only one DVH constraint is no longer satisfied in comparison with the original plan. With the kV scenario all criteria are still passed, so for these two scenarios plan acceptability is not markedly compromised by imaging. In contrast to that, scenario 4 results in a notable number of cases where the DVH constraints are no longer met, for the parotid glands the mean value exceeds the required 25 Gy for eight patients more than in the original plan. Nearly all DVH criteria are exceeded, some frequently, for this scenario leading to an entity of 20 cases, in which the given constraints are no longer satisfied. Obviously, fewer such cases can be found for the daily TBL scenario.

The mean values in Table 5 confirm these findings, scenario 2 and 3 showing only minor differences in comparison with the original plan. The daily kV imaging on average results in a smaller dose amount than the realistic scenario. Again, the table illustrates the marked influence of the IBL CBCTs on sparing of OARs, partly with an additional dose of up to a whole fraction dose. The values of scenario 5 range between those of scenario 2 and 3 and scenario 4. As expected, the statistical comparison results in clear significances (*p*-values < 0.001), in which the kV scenario results in a significant lower dose than the scenario considering the actual imaging for all parameters.

**Table 5:** Mean values ±standard deviation and range of the DVH criteria and NTCP results for the different scenarios

Organ	Criteria/endpoint	Original plan	Scenario 2	Scenario 3	Scenario 4	Scenario 5
Spinal chord	D2% < 42 [Gy]	32.7 ± 3.8	32.9 ± 3.8	32.8 ± 3.8	34.0 ± 3.8	33.5 ± 3.8
		23.3–41-3	23.4–41.5	23.3–41.5	24.1–42.6	23.7–42.1
	Myelitis/Necrosis [%]	0.1 ± 0.4	0.1 ± 0.4	0.1 ± 0.4	0.1 ± 0.5	0.1 ± 0.5
		0–2	0–2	0–2	0–2	0–2
Right Parotid gland	Mean < 25 [Gy]	19.3 ± 4.6	19.5 ± 4.6	19.4 ± 4.6	20.6 ± 4.8	20.0 ± 4.7
		9.5–25.4	9.6–25.5	9.6–25.6	10.7–27.3	10.2–26.5
	V20Gy < 60 [%]	35.0 ± 14.8	35.5 ± 15.1	35.3 ± 15.0	38.2 ± 16.4	36.7 ± 15.6
		0.0–57.9	0.0–59.3	0.0–58.3	0.0–63.0	0.0–60.9
	Xerostomia [%]	0.5 ± 0.8	0.5 ± 0.8	0.5 ± 0.8	0.8 ± 1.2	0.7 ± 1.0
		0–3	0–3	0–3	0–5	0–4
Left Parotid gland	Mean < 25 [Gy]	20.7 ± 4.0	21.1 ± 4.0	20.9 ± 4.0	22.2 ± 4.2	21.6 ± 4.1
		12.6–25.6	12.8–25.7	12.7–25.6	13.6–27.3	13.2–26.5
	V20Gy < 60 [%]	41.0 ± 11.2	41.7 ± 11.5	41.3 ± 11.3	44.5 ± 12.5	43.1 ± 11.9
		17.8–56.8	18.0–57.4	17.9–57.0	18.9–61.3	18.4–59.1
	Xerostomia [%]	0.6 ± 0.9	0.6 ± 0.9	0.6 ± 0.9	1.1 ± 1.3	0.8 ± 1.0
		0–3	0–3	0–3	0–5	0–4
Larynx	D2% < 63 [Gy]	54.8 ± 5.2	55.2 ± 5.2	55.0 ± 5.2	56.5 ± 5.3	55.8 ± 5.2
		43.8–63.4	44.1–63.8	43.9–63.5	45.5–65.1	44.7–64.3
	Mean < 44 [Gy]	34.4 ± 4.7	34.8 ± 4.7	34.5 ± 4.8	36.1 ± 4.8	35.4 ± 4.8
		18.2–42.9	19.4–43.3	18.3–43.0	19.5–44.8	19.0–43.9
	Edema [%]	11.2 ± 8.2	11.8 ± 8.5	11.4 ± 8.3	15.5 ± 10.3	12.7 ± 9.4
		0–30	0–32	0–31	0–37	0–34
Vocal chords	D2% < 25 [Gy]	22.0 ± 2.2	22.3 ± 2.3	22.1 ± 2.2	23.7 ± 2.2	23.0 ± 2.2
		17.5–26.0	17.7–26.3	17.6–26.1	20.0–27.8	18.8–26.9

Table 5 also lists the mean values of the NTCP for the considered endpoints. The only relevant probability (values over 1%) refers to the occurrence of Larynx edema. Even for the original plan NTCP amounts over 10%, which increases to over 15% by the daily use of IBL CBCTs.

### Positioning uncertainties

**Fig. 4:**
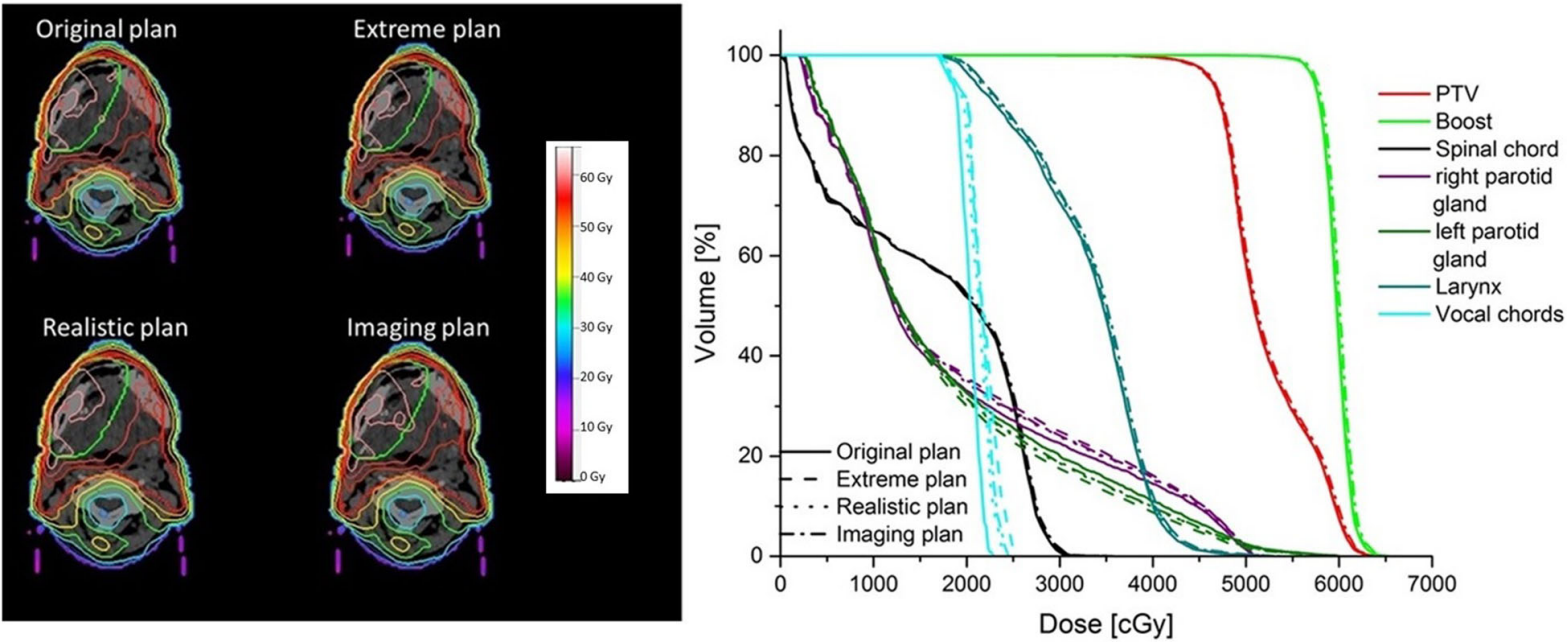
Example Dose distribution and DVH of one patient for all scenarios; same patient as in Figs. 1, 2 and 3

The visible inspection of the dose distributions and DVHs for all scenarios reveals only minor differences between the scenarios. Merely the combined scenario (imaging and positioning) leads to an expansion of the 100% isodose within the target volume (Fig. 4). Relating to OAR sparing, the right parotid gland and especially the vocal chords are best spared in the original plan, whereas the sparing of the left parotid gland is increased with the positioning uncertainties. However, for the whole collective the positioning errors result in a raised number of DVH constraints that are exceeded in comparison with the original plan (Table 6), especially for the parotid glands and the vocal chords.

**Table 6:** Number of exceedings of the considered planning criteria

Organ	Criteria	Original plan	Extreme plan	Realistic plan	Imaging plan
Spinal chord	D2% < 42Gy	0	1	0	0
Parotid glands	Mean < 25Gy	4	9	7	8
	V20Gy < 60%	0	2	1	2
Larynx	D2% < 63Gy	2	2	2	3
	Mean < 44Gy	0	0	0	0
Vocal chords	D2% < 25Gy	2	9	4	5
Total		8	23	14	18

In general, the same effects are observed for the realistic and the extreme plans, but in such a way that they are more pronounced for the extreme plan. This is not surprising, because the realistic plan is actually a weighted average of the extreme and the original plan, and should hence fall in between the two. It is also clear that due to the additional imaging dose the imaging plan contains more dose than the realistic scenario.

For the whole collective the mean values of the DVH constraints relating to sparing of OAR (Table 7) show a predominantly decreased sparing as a result of positioning errors, which is statistically significant for all cases besides the left parotid gland, with especial small *p*-values for the spinal chord and the vocal chords (< 0.001). Solely the left parotid gland is better spared with positioning inaccuracy (original plan: 19,82 Gy vs. extreme plan: 19,41 Gy). While the differences for the larynx are minor, the average values of the spinal chord and the right parotid gland differ about 1 Gy between original and extreme scenario, for the vocal chords even about 2 Gy (one whole fraction dose). As expected, the imaging scenario results for all cases in a significantly decreased sparing of OAR.

**Table 7:** Mean values ±standard deviation and range of the quality metrics, DVH criteria and NTCP results for the different scenarios. HI: Homogeneity Index, CI: Conformity Index, UR: Underdose rate, OR: Overdose rate, GI: Gradient Index

Organ	Criteria/endpoint	Original plan	Extreme plan	Realistic plan	Imaging plan
V95%	≥95 [%]	90.95 ± 6.43	90.01 ± 6.21	90.58 ± 6.20	91.62 ± 5.96
		72.01–99.08	74.09–97.93	74.4–98.31	74.50–98.39
HI	the smaller the better	0.19 ± 0.14	0.19 ± 0.14	0.19 ± 0.14	0.19 ± 0.14
		0.07–0.89	0.07–0.89	0.07–0.89	0.07–0.89
CI	the closer to 1 the better	0.62 ± 0.15	0.63 ± 0.14	0.63 ± 0.14	0.62 ± 0.15
		0.20–0.87	0.23–0.87	0.22–0.87	0.20–0.87
UR	the closer to 1 the better	0.91 ± 0.06	0.90 ± 0.06	0.91 ± 0.06	0.92 ± 0.06
		0.72–0.99	0.74–0.98	0.74–0.98	0.75–0.98
OR	the closer to 1 the better	0.69 ± 0.18	0.70 ± 0.18	0.70 ± 0.18	0.69 ± 0.19
		0.21–0.96	0.24–0.97	0.23–0.97	0.20–0.96
Spinal chord	D2% < 42 [Gy]	32.6 ± 3.9	33.6 ± 3.9	33.1 ± 3.9	33.3 ± 3.8
		23.0–41.2	25.9–42.5	24.7–41.5	24.8–41.8
	Myelitis/Necrosis [%]	0.3 ± 0.7	0.3 ± 0.8	0.3 ± 0.7	0.3 ± 0.8
		0.0–4.0	0.0–4.6	0.0–4.3	0.0–4.5
Right Parotid gland	Mean < 25 [Gy]	19.0 ± 4.6	20.6 ± 4.8	19.9 ± 4.6	20.2 ± 4.6
		9.5–25.3	9.7–28.9	9.6–27.4	9.8–27.6
	V20Gy < 60 [%]	34.0 ± 14.6	39.8 ± 15.7	37.5 ± 14.8	38.1 ± 15.1
		0.0–57.8	0.0–64.9	0.0–60.8	0.0–62.2
	Xerostomia [%]	0.3 ± 0.4	0.7 ± 1.2	0.5 ± 0.9	0.6 ± 0.9
		0.0–1.8	0.0–5.0	0.0–3.5	0.0–3.6
Left Parotid gland	Mean < 25 [Gy]	19.8 ± 3.7	19.4 ± 4.3	19.6 ± 4.0	19.9 ± 4.1
		12.6–24.4	11.0–26.4	11.7–25.6	11.9–25.8
	V20Gy < 60 [%]	41.1 ± 10.9	40.7 ± 12.6	40.9 ± 11.6	41.7 ± 11.9
		17.4–54.8	11.6–59.1	14.9–57.5	15.1–58.3
	Xerostomia [%]	0.3 ± 0.4	0.4 ± 0.5	0.4 ± 0.5	0.4 ± 0.5
		0.0–1.3	0.0–2.3	0.0–1.9	0.0–2.0
Larynx	D2% < 63 [Gy]	54.4 ± 5.1	54.4 ± 5.2	54.4 ± 5.2	54.7 ± 5.2
		43.1–63.2	44.5–62.9	44.0–62.8	44.3–63.1
	Mean < 44 [Gy]	34.4 ± 4.7	34.8 ± 4.7	34.7 ± 4.7	35.0 ± 4.8
		18.3–42.6	19.0–43.6	18.8–43.3	18.9–43.7
	Edema [%]	10.7 ± 7.5	11.4 ± 7.9	11.1 ± 7.8	11.9 ± 8.1
		0.1–27.6	0.1–29.3	0.1–28.7	0.1–30.1
Vocal chords	D2% < 25 [Gy]	21.5 ± 2.2	23.6 ± 2.4	22.6 ± 2.2	23.0 ± 2.2
		17.7–26.9	19.7–27.4	19.3–26.9	19.5–27.5

This is also reflected in the evaluation of NTCP. For all organs at risk NTCP values are lowest with the original scenario, especially for the larynx and the right parotid gland (Table 7). For these two organs pair-wise tests are significant (Larynx: original scenario vs. realistic scenario: *p* = 0.021, right parotid gland: original scenario vs. realistic scenario: *p* = 0.031).

For the examination of the influence of positioning errors on plan quality, measures of quality regarding PTV coverage are also considered (Table 7). The V95%, which is an important marker for the clinical routine, shows just minor deteriorations of target coverage as a result of positioning errors (differences of about 1% in comparison to the original plan). Homogeneity and conformity are also little affected by potential positioning errors for this collective. Although those measures of quality show just minor differences between the scenarios the statistical analysis results in significances for most cases (*p*-values< 0.03).

**Fig. 5:**
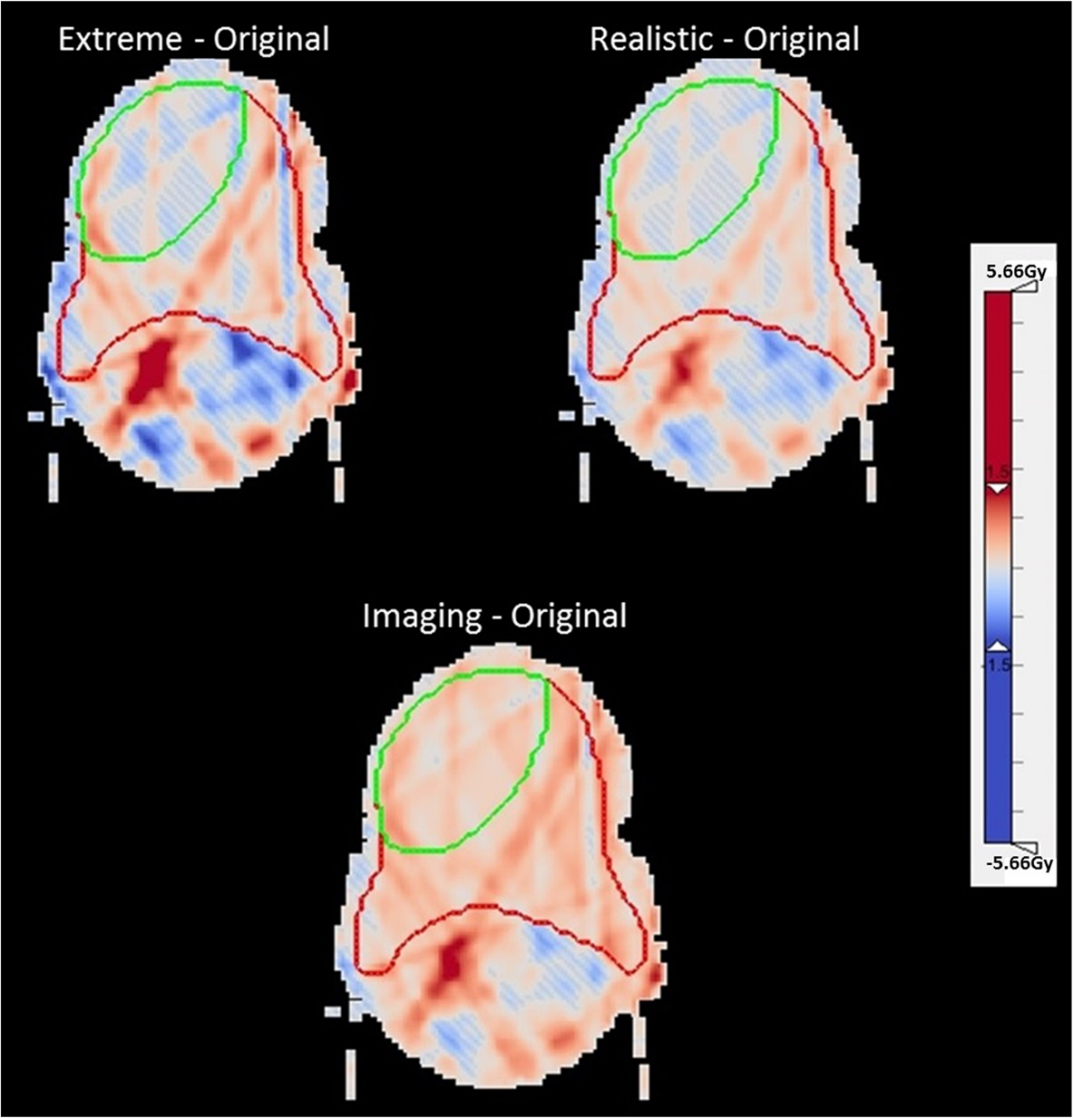
Example dose differences (cold and hot spots) for the three scenarios in comparison with the original plan

 Figure 5 illustrates the dose deviations in terms of “hot spots” (red) and “cold spots” (blue) of the different scenarios in comparison to the original plan. Over- and underdosages are of the same magnitude, in general the positioning errors result in balanced shifts for all directions. The additional imaging dose leads to a marked expansion of the area with positive dose differences.

For single cases even the realistic scenario shows dose differences of over 10 Gy (Table 8). The percentage of points with less than 2% and 3% dose differences yields values of over 90% for all scenarios. The 1% pass rates of the realistic and imaging scenarios are also still about 90% on average, whereas this is reduced for the extreme scenario to 83.8%. For all cases the large range shows that there are individual plans with extreme outliers.

**Table 8:** Mean values ± standard deviations and range of the dose differences and pass rates for the different scenarios in comparison with the original plan. “hot spot”: points of overdosage in comparison to the original plan, “cold spot”: points of underdosage in comparison to the original plan

Metric	Extreme	Realistic	Imaging
“cold spot” dose differences [Gy]	7.38 ± 3.53	4.61 ± 2.29	4.33 ± 2.30
	0.9–18.1	0.7–10.3	0.5–10.1
“hot spot” dose differences [Gy]	7.05 ± 3.85	4.37 ± 2.41	4.65 ± 2.41
	1.1–22.3	0.7–12.3	0.9–12.5
Pass rate for 3% local dose difference [%]	95.76 ± 3.94	98.09 ± 2.41	98.10 ± 2.37
	84.34–100	90.82–100	90.96–100
Pass rate for 2% local dose difference [%]	92.39 ± 5.79	96.08 ± 3.92	96.14 ± 3.90
	78.94–100	86.56–100	86.99–100
Pass rate for 1% local dose difference [%]	83.80 ± 8.50	89.82 ± 7.27	89.29 ± 7.20
	64.58–99.66	74.09–99.96	73.58–99.98

## Discussion

### Interpretation

Currently there are numerous studies that deal with imaging dose in radiation therapy. However, in large parts they focus on the additional imaging dose itself, especially for the comparatively newer kV modalities [6–13]. Amer et al. [7] examined CBCT skin doses of an Elekta Synergy X-Ray System. They measured doses of about 1.6 mGy in the head-&-neck region. This can be confirmed by our findings of about 3–9 mGy for one head and neck CBCT [14]. In [14] we also give magnitudes for MV CBCTs, the IBL results in 34–62 mGy, while imaging using the treatment beam line provides about 80 mGy per CBCT in the head area. Further studies, in which imaging doses are explicitly calculated on the planning CT, also focus primarily on the kV modality. Alaei et al. [17] found an additional dose of about 30–40 mGy for 35 fractions head and neck treatment when using daily kV CBCT, which is comparatively less dose than our results for head and neck treatment show.

At our department the use of three energy-matched linacs with different imaging modalities leads to variations of set-up verifications regarding frequency and technique. While it is already shown in several previous tests, this study confirms that the actual performed imaging has little influence on plan quality. However, set-up verification is not done daily, so that uncorrected positioning errors could also affect plan quality. This trade-off between additional imaging dose and dose smearing was meant to be analyzed more accurately.

For daily imaging we can assume that no dose smearing comes into effect as positioning errors can be neglected. In a previous study we confirmed that for all imaging modalities set-up verifications can be performed with comparable precision [20].

Daily imaging using kV CBCTs just contributes a minor additional dose. This scenario is highly recommended for the clinical routine. Beside the low influence of the imaging dose on the plan quality, kV imaging yields the best image quality, so that image fusion is not restricted to bony structures. However, not every institution is equipped with a kV modality, so it should also be analyzed, if daily imaging is advisable for MV energies, too.

The Siemens specific IBL daily CBCT scenario leads to a very high dose amount of up to one additional fraction dose. Numerous DVH constraints are exceeded and NTCP is markedly increased, so that most of the plans are clinically not acceptable anymore. Caution is especially advised for the interpretation of the name of this modality. It is marketed as “kView”, what pretends to be a kV-like modality. However, it should be kept in mind that the image beam line is a MV modality and the results show the negative effects of the additional dose although it is lower in comparison to the treatment beam line.

The use of daily CBCT with the TBL would result in inacceptable dose contributions. If the volumetric recordings are partly or completely replaced by planar images, this offers an acceptable alternative for daily 6 MV set-up verification. The dose can be even more reduced by the application of the IBL energy within this scenario.

If daily volumetric MV imaging is still required, it is also feasible to calculate the additional imaging dose already in the planning process. This leads to a realistic approximation and sparing of organs at risk and target coverage can be optimized in advance with the integration of the additional dose.

One point regarding frequency and technique of set-up verifications that should not be neglected is the treatment time. Imaging to control the patient’s position is time consuming, which plays a central role for the schedule of the clinical routine. This point is also relevant for the patients. Long lay times should be avoided, especially for patients in pain but also to prevent from the opportunity of more patient-movements before the treatment starts. Generally, daily set-up verifications for every single patient prove to be difficult. That is why the influence of potential positioning errors on plan quality should not be neglected.

We simulated an extreme case for the absence of verification images as well as a realistic case, where an exact positioning was supposed for those fractions with set-up verifications.

Sparing of OAR is markedly decreased due to positioning uncertainties, which is significant for the whole collective. This is also reflected in the NTCP modelled for clinical endpoints.

One last step was the creation of an imaging plan as an extension of the realistic case with the associated imaging dose. This serves as the actual case, where both effects are combined under real conditions. With the additional imaging dose, the decrease of OAR sparing due to positioning errors for this collective continues to deteriorate.

This study shows that steady set-up verifications are reasonable and indispensable. The additional imaging dose can play a minor role, especially by the use of lower energy modalities. Daily imaging using kV energies was shown to have the least dosimetric impact. However, as the time schedule and technical circumstances do not always allow for applying this scenario, frequency and technique of the positioning verifications should be adapted to the relevant requirements. The thermoplastic masks for H&N treatments already offer a quite precise positioning. Generally, the IGRT concept should be a more individual decision. We saw large ranges in this study, an adaption on individual experiences for every patient after the first few fractions should be preferred compared to a fixed schedule.

### Limitations

All positioning uncertainties considered in this study result in an incorrect positioning of the patient. No anatomical deformations were included. This would be a more important point when considering prostate treatment, for example. However, in the head and neck area there is less proper motion of the organs relative to the bony structures to be accounted for. Moreover, considering only the patients’shifts does not yield any difficulties of changing OAR and PTV volumes, deformable registration and DVH dose accumulation. Besides, this approach allowed us to include set-up information from planar imaging rather than just CBCT in the analysis, which is clinically more relevant.

The usage of safety margins to avoid underdosage of the target volume due to positioning errors is standard nowadays. In our institution margins are adapted to potential positioning errors, but not necessarily in conjunction with an explicitly contouring of a CTV. The PTV is contoured directly with a safety margin of 5–10 mm to the tumor area. So in this study we returned the analysis only to the planning target volume, the influence of positioning errors on the clinical target volume could not be evaluated. In the framework of such an evaluation it would also be possible to determine the tumor control in terms of a TCP (Tumor Control Probability) analysis. However, in clinical reality it is the PTV that is normally considered for target coverage in many institutions – in our clinic, target coverage is considered acceptable as long as 95% of the PTV is covered by the 95% prescription isodose (However, this is not always feasible due to constraints on the OARs). Nonetheless, with an IGRT scenario featuring regular or even daily imaging the safety margins can be chosen considerably smaller. This leads automatically to a marked change in the initial dose distribution and indicate a clear benefit from using small meshed IGRT [33]. Beyond margin reduction, regular image-guidance can also allow for detecting pronounced anatomical changes and thus trigger a re-planning for the patient.

## Conclusion

Plan quality is only marginally affected by the application of set-up verifications with kV energies. So this modality can be used for the clinical routine without reservation, even daily. If no kV modality is available, daily volumetric verification images should be avoided with MV energies without including the additional dose amount to the treatment plan beforehand. Within this energy a scenario with mostly planar imaging should be preferred.

Dose smearing due to positioning errors for this collective mainly resulted in a decrease of OAR sparing. Target coverage also suffered from the positioning inaccuracies, especially for individual patients. The difference between the extreme scenario, in which the omission of any set-up verification is simulated, and the realistic scenario, in which those fractions with imaging are considered, shows the large benefit of regular positioning checks.

Taking into account both examined effects the relevance of an extensive IGRT is clearly present, even at the expense of additional imaging dose and time expenditure.

## Abbreviations

CBCT: Cone Beam Computer Tomography
CERR: Computational Environment for Radiotherapy Research
CI: Conformity Index
CT: Computed Tomography
CTV: Clinical Target Volume
DICOM: Digital Imaging and Communication in Medicine
DVH: Dose Volume Histogram
H&N: Head and Neck
HI: Homogeneity Index
IBL: Imaging Beam Line
IGRT: Image Guided Radiation Therapy
LKB: Lymann Kutcher Burrmann
MU: Monitor Units
NTCP: Normal Tissue Complication Probability
OAR: Organ At Risk
OR: Overdose Ratio
PTV: Planning Target Volume
R&V: Record & Verify
TBL: Treatment Beam Line
TCP: Tumor Control Probability
TPS: Treatment Planning System
UR: Underdose Ratio
